# Supplementary figures and images for: Characterizing non-exponential growth and bimodal cell size distributions in fission yeast: An analytical approach
Source: PLoS Comput Biol. 2022 Jan 18;18(1):e1009793. doi: 10.1371/journal.pcbi.1009793 (PMC8797179; doi:10.1371/journal.pcbi.1009793)

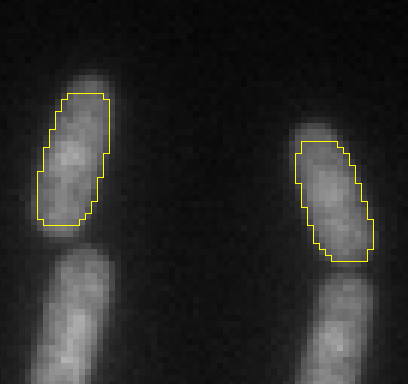

Supplement: S3 Fig — At division, the segmentation algorithm tends to cut old-pole tips. (TIFF) [file pcbi.1009793.s004.tiff]
