# Supplementary material for: Characterizing non-exponential growth and bimodal cell size distributions in fission yeast: An analytical approach
Source: PLoS Comput Biol. 2022 Jan 18;18(1):e1009793. doi: 10.1371/journal.pcbi.1009793 (PMC8797179; doi:10.1371/journal.pcbi.1009793)
Supplement: Attachment — Submitted filename: reference_list.pdf [file pcbi.1009793.s005.pdf]

References

1. Mitchison, J.M.; Nurse, P. Growth in cell length in the fission yeast Schizosaccharomyces pombe. *J. Cell Sci.* **1985**, *75*, 357–376.
2. May, J.W.; Mitchison, J.M. Pattern of polar extension of the cell wall in the fission yeast Schizosaccharomyces pombe. *Can. J. Microbiol.* **1995**, *41*, 273–277.
3. Baumgartner, S.; Tolic-Norrelykke, I.M. Growth pattern of single fission yeast cells is bilinear and depends on temperature and DNA synthesis. *Biophys. J.* **2009**, *96*, 4336–4347.
4. Cooper, S. Schizosaccharomyces pombe grows exponentially during the division cycle with no rate change points. *FEMS Yeast Res.* **2013**, *13*, 650–658.
5. Horváth, A.; Rácz-Mónus, A.; Buchwald, P.; Sveiczer, A. Cell length growth in fission yeast: an analysis of its bilinear character and the nature of its rate change transition. *FEMS Yeast Res.* **2013**, *13*, 635–649.
6. Pickering, M.; Hollis, L.N.; D'Souza, E.; Rhind, N. Fission yeast cells grow approximately exponentially. *Cell Cycle* **2019**, *18*, 869–879, doi:10.1080/15384101.2019.1595874.
7. Knapp, B.D.; Odermatt, P.; Rojas, E.R.; Cheng, W.; He, X.; Huang, K.C.; Chang, F. Decoupling of Rates of Protein Synthesis from Cell Expansion Leads to Supergrowth. *Cell Syst.* **2019**, *9*, 434–445.e6, doi:10.1016/j.cels.2019.10.001.
8. Neumann, F.R.; Nurse, P. Nuclear size control in fission yeast. *J. Cell Biol.* **2007**, *179*, 593–600.
9. Moreno, S.; Nurse, P. Regulation of progression through the G1 phase of the cell cycle by the rum1+ gene. *Nature* **1994**, *367*, 236–242.
10. Allard, C.A.H.; Opalko, H.E.; Liu, K.W.; Medoh, U.; Moseley, J.B. Cell size-dependent regulation of Wee1 localization by Cdr2 cortical nodes. *J. Cell Biol.* **2018**, *217*, 1589–1599, doi:10.1083/jcb.201709171.
11. Gerganova, V.; Bhatia, P.; Vincenzetti, V.; Martin, S.G. Direct and indirect regulation of Pom1 cell size pathway by the protein phosphatase 2C Ptc1. *Mol. Biol. Cell* **2021**, *32*, 703–711, doi:10.1091/mbc.e20-08-0508.
12. Martin, S.G.; Berthelot-Grosjean, M. Polar gradients of the DYRK-family kinase Pom1 couple cell length with the cell cycle. *Nature* **2009**, *459*, 852–856, doi:10.1038/nature08054.
13. Moseley, J.B.; Mayeux, A.; Paoletti, A.; Nurse, P. A spatial gradient coordinates cell size and mitotic entry in fission yeast. *Nature* **2009**, *459*, 857–860.
14. Opalko, H.E.; Nasa, I.; Kettenbach, A.N.; Moseley, J.B. A mechanism for how Cdr1/Nim1 kinase promotes mitotic entry by inhibiting Wee1. *Mol. Biol. Cell* **2019**, *30*, 3015–3023, doi:10.1091/mbc.E19-08-0430.
15. Rhind, N.; Ohira, M.J.; D'Souza, E.; Magner, M.; Mayhew, M.B.; Keifenheim, D.; Sun, X.-M.; Marguerat, S. Size-Dependent Expression of the Mitotic Activator Cdc25 Suggests a Mechanism of Size Control in Fission Yeast. *Curr. Biol.* **2017**, *27*, 1491–1497, doi:10.1016/j.cub.2017.04.016.
16. Navarro, F.J.; Weston, L.; Nurse, P. Global control of cell growth in fission yeast and its coordination with the cell cycle. *Curr. Opin. Cell Biol.* **2012**, *24*, 833–837, doi:10.1016/j.ceb.2012.10.015.
17. Patterson, J.O.; Rees, P.; Nurse, P. Noisy Cell-Size-Correlated Expression of Cyclin B Drives Probabilistic Cell-Size Homeostasis in Fission Yeast. *Curr. Biol.* **2019**, *29*, 1379–1386.e4, doi:10.1016/j.cub.2019.03.011.
18. Nurse, P.; Fantes, P.A. Cell cycle controls in fission yeast: a genetic analysis. In *The Cell Cycle*; John, P.C.L., Ed.; Cambridge University Press: Cambridge, 1981; pp. 85–

98.
19. Sveiczer, A.; Novak, B.; Mitchison, J.M. Mitotic control in the absence of cdc25 mitotic inducer in fission yeast. *J. Cell Sci.* **1999**, *112*, 1085–1092.
20. Horváth, A.; Rácz-Mónus, A.; Buchwald, P.; Sveiczer, Á. Cell length growth patterns in fission yeast reveal a novel size control mechanism operating in late G2 phase. *Biol. Cell* **2016**, *108*, 259–277, doi:10.1111/boc.201500066.
21. Sveiczer, A.; Novak, B.; Mitchison, J.M. The size control of fission yeast revisited. *J. Cell Sci.* **1996**, *109*, 2947–2957.
22. Nagy, Z.; Medgyes-Horváth, A.; Vörös, E.; Sveiczer, Á. Strongly oversized fission yeast cells lack any size control and tend to grow linearly rather than bilinearly. *Yeast* **2021**, *38*, 206–221, doi:10.1002/yea.3535.
23. Coudreuse, D.; Nurse, P. Driving the cell cycle with a minimal CDK control network. *Nature* **2010**, *468*, 1074–1079.
24. Schmoller KM The phenomenology of cell size control. *Curr Opin Cell Biol* **2017**, *49*, 53–58, doi:10.1016/j.ceb.2017.11.011.
25. Scotchman, E.; Kume, K.; Navarro, F.J.; Nurse, P. Identification of mutants with increased variation in cell size at onset of mitosis in fission yeast. *J. Cell Sci.* **2021**, *134*, doi:10.1242/jcs.251769.
26. Odermatt, P.D.; Miettinen, T.P.; Lemière, J.; Kang, J.H.; Bostan, E.; Manalis, S.R.; Huang, K.C.; Chang, F. Variations of intracellular density during the cell cycle arise from tip-growth regulation in fission yeast. *Elife* **2021**, *10*, 1–23, doi:10.7554/elife.64901.
27. Sveiczer, Á.; Horváth, A.; Buchwald, P. Is there a universal rule for cellular growth? – Problems in studying and interpreting this phenomenon. *FEMS Yeast Res.* **2014**, *14*, 679–682.
28. Mitchison, J.M. Growth during the cell cycle. *Int. Rev. Cytol.* **2003**, *226*, 165–258.
