# Supplementary material for: Characterizing non-exponential growth and bimodal cell size distributions in fission yeast: An analytical approach
Source: PLoS Comput Biol. 2022 Jan 18;18(1):e1009793. doi: 10.1371/journal.pcbi.1009793 (PMC8797179; doi:10.1371/journal.pcbi.1009793)
Supplement: Attachment — Submitted filename: Referee_response_PCB2021.pdf [file pcbi.1009793.s006.pdf]

Response to Referee 1

Thank you for your valuable comments and suggestions which have helped us greatly in improving the quality of this paper. Below we provide a point-by-point response to each comment. All modifications made in the revised manuscript are shown in blue.

1. *The introduction fails to adequately summarise what is known about how fission yeast control their size given that there is there is an extensive amount of literature that the authors have failed to properly introduce. A more detailed account of existing literature is required given the subject matter of the article. In a similar vein it is kind of inexplicable that the authors do not even cite Fantes'77 given it is the foundational study for growth and size control of fission yeast.*

Similarly, there is also a significant amount of work that describes the growth patterns of fission yeast as a combination of multiple linear (or one exponential, depending on the study) growth phases with a constant length period during mitosis and separation before division. Again, this is not really discussed at all in the introduction and the foundational studies including original account of these phenomena Mitchinson and Nurse'85 are not even cite, let alone discussed despite being highly relevant.

Response: In the revised manuscript, we have added many important references about the growth pattern and size control of fission yeast, including the foundational studies of Fantes'77 and Mitchinson and Nurse'85 (see pages 27-31). Moreover, we have added a discussion summarizing what is known about the growth pattern and size control of fission yeast in the Introduction (see pages 2-3).

2. *The authors state that the growth in "the elongation phase is assumed to be linear." This is misleading to the point of being wrong. There is very little evidence that the growth phase in fission yeast is a simple linear growth. The predominant idea is that elongation phase is bi-linear, i.e. it is constituted of two linear growth regimes with different growth rates separated by a rate change point. In reality this is very close to an exponential growth pattern and it is very difficult to distinguish these two possibilities experimentally.*

Response: Thank you for pointing out this mistake. In the introduction section of the revised manuscript, we have added some discussion summarizing what is known about the growth pattern of fission yeast, including exponential, linear, and bilinear models proposed in previous papers (see page 3). Moreover, we have emphasized that in practice, it is difficult to distinguish the exponential and bilinear growth patterns due to the stochasticity of growth dynamics and the relatively low temporal and spatial resolution of the data (see page 6).

3. *Regarding the authors statement that "the linear relationship between birth and division sizes are actually very weak" which "makes the inference of the parameter β highly unreliable". Sizer and adder models are more typically assessed using the relationship between birth size and Δvolume. These relationships have been published many times since originally described by Fantes in 1977 and have generally shown very robust correlations. This relationship is critical and must be shown for both the data and the models including the in an updated version of table 3.*

Response: In the revised manuscript, we have added the scatter plot of the birth size V_b versus the added size $V_d - V_b$ in the introduction part (see page 4, Figure 1(d)). Moreover, we have updated Table 3 by showing the experimental correlation coefficients between V_b and $V_d - V_b$ for all growth conditions, as well as the theoretical correlation coefficients based on model I and model II (see page 23, Table 3).

4. *The authors assume that there is a short increase in cell size prior to division and model this as an exponential growth rate with a higher exponent than that in the subsequent elongation phase. When a cell divides the new end forms a semi-sphere which pops out due to hydrostatic pressure. Is it not possible / likely that this increase in size in the fission phase the authors are modeling is due to expansion of the new ends due to cell division and hydrostatic pressure? If so there is no basis to the assumption this volume growth is exponential because the semi-sphere that pops out is presumably always approximately the same size and takes the same time. This needs to be investigated carefully and the model updated accordingly.*

Response: In the revised manuscript, we have clarified that the rapid increase of cell length in the reshaping phase is the result of the rounding off of the new ends due to hydrostatic pressure. Moreover, we have also explained why we assume exponential growth with a higher growth rate in this phase (see pages 6-7). According to our data analysis, for all growth conditions, the added size and the duration in the reshaping phase are highly variable. For example, for the YE medium at 34°C, the mean and standard deviation of the added length in the reshaping phase are given by 1.590 ± 0.645 μm with a high coefficient of variation (CV) of 40.6%; the mean and standard deviation of the duration in the reshaping phase are given by 9.040 ± 3.274 minutes with a high CV of 36.2%. In our model, the generalized added size $\Delta_1 = V_d^\alpha - V_s^\alpha$ in the reshaping phase has an Erlang distribution with shape parameter N_1, whose CV is given by $1/\sqrt{N_1}$. When N_1 is small, the CV of the generalized added size is very large. This is consistent with experimental data.

5. *Throughout the authors refer to cell size which is generally term that encompasses many cellular parameters. In reality the authors are only looking at cell area, rather than dry mass, buoyant mass, total protein content etc. The text should be updated accordingly. Moreover, it would be far more useful if the authors converted their area estimates to volume for more ready comparison between studies.*

Response: In the revised manuscript, we have emphasized that cell size in the main text is referred to as either cell length, cell area, or cell volume (see page 5). Moreover, to make our results easily compared to the literature, we have also converted cell area data to cell length data by using the information of mean cell diameter for each growth condition, which can be estimated from the fluorescence images provided to us by the authors of Reference [4] (see pages 17-25).

Response to Referee 2

Thank you for your valuable comments and suggestions which have helped us greatly in improving the quality of this paper. Below we provide a point-by-point response to each comment. All modifications made in the revised manuscript are shown in blue.

1. *The reference list contains rather few items and their selection seems to be strange. Please note that there is a huge number of published papers from the last 30 years concerning cellular growth and size control in fission yeast as well as their molecular background (I upload a list, which is also far from full). By contrast, among the authors 33 items, only 7 deals very strictly with the experimental background of this theoretical work. In the other papers I attach, there are lots of data for size distributions, growing patterns, size control strength in fission yeast cultures, moreover, proteins and genes involved in these phenomena, etc. The authors should refer to a much broader list and also explain why they chose exclusively the data of Nakaoka and Wakamoto (PLOS Biol, 2017) for their model fitting.*

Response: Thank you for providing us with so many important references about the growth pattern and size control of fission yeast. In the revised manuscript, we have added all of them and some other related papers into our reference list (see pages 27-31). Moreover, we have also explained why we choose exclusively the data in the Nakaoka-Wakamoto paper for our model fitting (page 17). In fact, this data set contains high throughput data of the whole time series of thousands of individual cells followed over many cell cycles, instead of data at some particular time points such as data of cell sizes at birth, septation, and division. The monitoring of the whole time series allows an accurate inference of all model parameters as well as a deeper understanding of the full cell cycle dynamics. We have not been able to find another cell lineage data of fission yeast with so many data points.

2. *A problem (the smaller one) with the Nakaoka-Wakamoto paper (and evidently with the simulations) is that cell size is given as "area" in dimension of micrometer^2. This is difficult to measure, rarely used, and therefore the numbers practically do not say anything to specialists of this field. Instead, mainly "cell length" (in microns) or perhaps "cell volume" (in femtoliters) are the commonly used parameters for fission yeast cell size, which are generally given in experimental papers, therefore simulations of these parameters could be easily compared to literature data.*

Response: In the revised manuscript, to make our results easily compared to the literature, we have converted cell area data to cell length data by using the information of mean cell diameter for each growth condition, which can be estimated from the fluorescence images provided to us by the authors of the Nakaoka-Wakamoto paper (see pages 17-25).

3. *The much larger problem with the simulations and the experimental data behind is the following. What the authors call "fission phase" (explained in their Fig. 2), is generally thought to belong to the next cell cycle, i.e., what they call Vs is generally called Vd, and Vb is also generally thought to be quite different. When the primary septum starts to become degraded, then we consider that the mother cell has divided into two progenies. Defining the birth and division times and sizes the way given in this manuscript once seems to be difficult to be defined precisely. Moreover, the authors study a size*

range different from the conventionally used one. Finally, I have a feeling that even the bimodal size distribution might be an artefact of the incorrectly positioned division times. To my mind, it is not a general view that size distribution in S. pombe cultures were bimodal, even I cannot fine the bimodal experimental histograms in the Nakaoka-Wakamoto paper. So, I cannot accept the sentence from the Author summary saying that "two characteristic cell sizes exist". This is probably the main point needed to be either discussed in detail or fixed in a revised version!

Response: In the revised manuscript, we have discussed in detail the differences between our definition and the definition in previous papers. Moreover, we have also explained why we include the reshaping phase (previously referred to as the fusion phase) into the former cell cycle instead of the next (see pages 8-9). Indeed, the bimodal cell size distribution of fission yeast is rarely mentioned in the literature. The possible reason for this fact is stated in the introduction part (see page 3).

4. *There is also another bombast, but incorrect sentence in the Author summary saying that "we construct the first mathematical model of this organism".*

Response: In the revised manuscript, we have revised this sentence (see page 2).

5. *To my mind, whether "beta = 2 corresponds to the timer strategy" (page 3) depends on the growth mode; it is correct in case of a pure exponential growth pattern only. Moreover, this parameter beta is often called the strength of size control in the literature. By contrast, the author define an alpha parameter (pages 5, 6), which is called the strength of size control. Will the authors give us the connection between these alpha and beta parameters?*

Response: In the revised manuscript, we have found a simple formula connecting the size control strength α in our model with the size control strength β in the conventional model under the assumption of symmetric division (see page 7). In fact, they are related by $\beta = 2^{1-\alpha}$ (see Supplementary Section 1 for the proof). Moreover, we have also proved that "$\beta = 2$ corresponds to the timer strategy" does not depend on the growth pattern and also holds when the growth is non-exponential (see Supplementary Section 1 for the proof).

6. *In the legend to Fig. 1, the phrase "length of each generation" should be replaced by "generation time".*

Response: Fixed as suggested (see page 4). Thank you.

7. *Please note that even if a fission yeast cell divides asymmetrically, the diameters of the progenies are usually equal, therefore there is a mistake in Fig. 2b.*

Response: Fixed as suggested (see page 5, Figure 2(b)). Thank you.

8. *The authors suppose that growth is exponential in the elongation phase (characterized by a g_0 parameter), although they mention that "in some previous papers it is assumed to be linear" (page 5).*

By contrast, this debate has not been resolved by now, and bilinear growth was also found in a paper in 2021. This should be discussed in the paper.

Response: In the revised manuscript, we have added some discussion summarizing what is known about the growth pattern of fission yeast, including exponential, linear, and bilinear models proposed in previous papers (see page 3). Moreover, we have emphasized that in practice, it is difficult to distinguish the exponential and bilinear growth patterns due to the stochasticity of growth dynamics and the relatively low temporal and spatial resolution of the data (see page 6).

9. *The authors suppose that growth is also exponential in the fission phase (characterized by a $g_1 > g_0$ parameter). Besides my point 2.), there is another problem here. It is generally assumed that in this stage cell elongation is not really growth, but it is rather the rounding off of the new cell ends from the septum, which is driven by mechanical forces mainly. The duration of this phase is very variable and it probably depends on geometrical and osmotic factors. The way how cells elongate here shows abnormalities, therefore this part is often omitted from cell length growth studies. The authors should explain their exponential hypothesis.*

Response: In the revised manuscript, we have clarified that the rapid increase of cell length in the reshaping phase is the result of the rounding off of the new ends due to hydrostatic pressure. Moreover, we have also explained why we choose to include that phase into our modelling and why we assume exponential growth with a higher growth rate in this phase (see pages 6-7). Indeed, the added size and the duration in the reshaping phase are highly variable. For example, for the YE medium at 34°C, the mean and standard deviation of the added length in the reshaping phase are given by 1.590 ± 0.645 μm with a high coefficient of variation (CV) of 40.6%; the mean and standard deviation of the duration in the reshaping phase are given by 9.040 ± 3.274 minutes with a high CV of 36.2%. In our model, the generalized added size $\Delta_1 = V_d^\alpha - V_s^\alpha$ in the reshaping phase has an Erlang distribution with shape parameter N_1, whose CV is given by $1/\sqrt{N_1}$. When N_1 is small, the CV of the generalized added size is very large. This is consistent with experimental data.

10. *The model supposes that the cell cycle consists of effective stages (N, N0, N1) and transitions from one to the next, but this is obscure. Although some cell cycle transitions are cytologically known, like the G1/S, G2/M and the metaphase/anaphase transitions, the authors should give us clear ideas on what they are talking about. For example, they mention some mysterious "division proteins", but their examples (Cdc13, Cdc25, Cdr2) are probably all required for the same G2/M transition. Please also note that these proteins are often regulated post-translationally, therefore their activities matter rather than their levels (page 6). The value of these N parameters is also interesting. In Table 2, it is given somewhere between 16 and 55, which seems to be unexpectedly large. Moreover, if it characterizes the number of cell cycle stages, how might it depend on the culturing techniques (medium, temperature) applied?*

Response: First, in the revised manuscript, we have emphasized that the effective cell cycle stages in our model do not directly correspond to the four biological cell cycle phases (G_1, S, G_2, and M) or the three growth phases (elongation, septation, and reshaping). Rather, a cell cycle phase or a growth phase

corresponds to multiple effective cell cycle stages (see page 7).

Second, we have explained in detail that it is widely believed that the accumulation of regulators upstream of Cdk1, such as Cdr2, Cdc25 and Cdc13, to a critical threshold is used to promote mitotic entry and trigger cell division, a strategy known as activator accumulation mechanism (see, for example, page 3 of Reference [54]). Here it is indeed the levels of Cdr2, Cdc25, or Cdc13 that matters. For Cdr2, please refer to page 3 of Reference [54]; for Cdc25, please refer to page 1 of Reference [57]; for Cdc13, please refer to page 1382 of Reference [58]. Moreover, we have emphasized that the N effective cell cycle stages in our model can be understood as different levels of the key protein that triggers cell division (see page 7).

Finally, we have discussed why the number N of effective cell cycle stages depend on the culturing condition (strain, medium, temperature) applied (see page 8).

11. *I think that r1 = N1/N, so there is a wrong lowercase "n" in page 9.*

Response: Fixed as suggested (see page 11). Thank you.

12. *In Fig. 3. it should be more clearly indicated which parameter set gives the best fit to Fig. 1.*

Response: This information is provided in Table 2 (34C YE medium).

13. *The correlation coefficient between V_b and V_d may really reflect the size control in the population. However, it is not clear why should raise them to the parameter alpha and calculate the correlation coefficient this way (page 15). What is the physiological meaning of this correlation coefficient?*

Response: In the revised manuscript, we have explained why we computed the correlation coefficient of V_b^α and V_d^α, rather than the correlation coefficient of V_b and V_d (see page 17).

14. *Are the experimental data in Figs. 5 and 6 and Table 3 from reference [11]? It should be indicated then, however, I could not find these bimodal distributions in that paper, even not in its supplementary material.*

Response: Yes, the experimental data used in Figure 5, Supplementary Figure 2 (Figure 6 in the previous version), and Tables 2-3 are from the Nakaoka-Wakamoto paper. In the revised manuscript, we have emphasized this point in the captions of Figure 5 and Table 3 (see pages 21 and 23). The cell size distribution of lineage measurements was not discussed in the Nakaoka-Wakamoto paper. That is why these bimodal distributions cannot be found in that paper.

15. *The general conclusions for the model fittings seem to be quite correct concerning the sizer-like behaviour and the durations of the cell cycle phases (page 19), but some literature data should be given for comparison here. The way the authors repeat these findings in the Discussion (points (iii) and (iv)) is incorrect.*

Response: In the revised manuscript, we have compared our estimated parameters with the values in previous papers. Specifically, we have shown that the estimated values of the mean septation length, the proportion of the elongation phase, as well as the duration and length increase in the reshaping phase are all consistent with the literature (see page 24). Moreover, we have revised the statement in the conclusions and discussion part (see page 26).

16. *Speaking about "slow growth" in the elongation phase and "rapid growth" in the fission phase (Discussion) is meaningless. See also 9.).*

Response: We totally agree with you that cell volume may not change in the reshaping phase. However, cell length indeed increases rapidly in this phase. In the revised manuscript, we have changed "rapid growth" to "rapid elongation" in the conclusions and discussion part (see page 25).

17. *We may say that size control seems to be stronger in EMM than in YE medium. However, speaking about strong size control in EMM and weak one in YE (Discussion) is meaningless.*

Response: we have revised the statement in the conclusions and discussion part (see page 26).

18. *Although I have concerns about the model described in this paper, I admit that the simulations were perfectly fitted to the experiments, both in examining size distributions and size control parameters (Figs. 5, 6, Table 3). I can imagine a third method to study how adequate the model is. In the literature the authors may find cell length growth patterns for either representative individual fission yeast cells or for hypothetical "average" cells, measured in similar conditions. Could the authors fit their model to some cell length growth patterns? To my mind, such a presentation might really be convincing.*

Response: In the revised manuscript, to make our results easily compared to the literature, we have converted cell area data to cell length data by using the information of mean cell diameter for each growth condition, which can be estimated from the fluorescence images provided to us by the authors of the Nakaoka-Wakamoto paper (see pages 17-25). In order to fit our theory to experiments, we need high throughput time-course data of fission yeast cell size across many generations. We could not find other fission yeast lineage data in the literature that we can use to do inference and contrast with our present results based on the Nakaoka-Wakamoto paper.

Response to Referee 3

Thank you for your valuable comments and suggestions which have helped us greatly in improving the quality of this paper. Below we provide a point-by-point response to each comment. All modifications made in the revised manuscript are shown in blue.

1. *The paper claims that the good fit of the model to data provides support for the model assumptions. However, they do not show any alternative simpler models that could fails capturing the bimodality of the data. Maybe, best fit results for one stage, two stage or alternative 3 stage models could be shown? Also, the point of the two models (model I and II) is very unclear, as the authors do not make any attempt to rule one out. If they are both good, I suggest to move model II results, completely to Supplement and just have a paragraph on that in the main paper. I appreciate the analytical results are cool and makes inference easier, but they are not that relevant to the science.*

Response: In the revised manuscript, we have fitted the experimental cell size distribution to the one-stage model with only the elongation phase and the two-stage model with only the elongation and septation phases, by using the same inference method. Both simpler models fail to capture the unusual shape of the cell size distribution (see pages 22-23 and Figure 5(d)). The one-stage model always predicts a unimodal distribution; the two-stage model predicts a unimodal distribution for EMM and a bimodal one for YE. While the two-stage model excellently captures the left peak of the distribution, it fails to reproduce the right peak due to the neglection of the reshaping phase. This suggests that our model is the simplest model that can describe the lineage data of fission yeast.

In the revised manuscript, we have not moved all model II results to Supplementary Material due to the following reason. While both model I and model II capture data excellently, model I gives rise to lower estimates of α, N_1, and w_1, as well as higher estimates of N, a, and g_1. This means that the usage of model I may lead to incorrect predictions. For example, since model I leads to an underestimate of the strength α of size control, it may occur that a sizer-like $(\alpha > 1)$ control strategy is mistaken for a near-adder $(\alpha \approx 1)$ control strategy. Therefore, all biological discussions should be based on the the parameters of model II since this is biologically more realistic and thus its parameter estimates are more reliable. We have emphasized this point in the revised manuscript (see pages 23-24). Due to the importance of model II, we did not move the relevant results to Supplementary Material.

2. *Instead of current Figure 6, what is more helpful is to turn some of the results in table II into graphs. What is happening to α as a function of growth rate, How is the fraction of non-growing phase changes as a function of cell cycle time? Here is some of the biologically relevant results that is now buried on a big table of numbers.*

Response: In the revised manuscript, based on the estimated parameters, we have investigated the dependence of the parameters N, α, w_0, and g_1 on the growth rate g_0 in the elongation phase (see page 23, Figure 6). In particular, we found a positive correlation between g_0 and N, a negative correlation between g_0 and α, a negative correlation between g_0 and w_1, and a positive correlation between g_0 and g_1. All these findings are analyzed in detail in the revised manuscript (see pages 24-25).

3. *There is an old and large literature that suggests Bi-linear growth in fission yeast and the concept of NETO, more recent data has confirmed exponential growth of mass but some changes of cell density (Fred Cheng papers). This could be briefly discussed in the introductions.*

Response: In the revised manuscript, we have added some discussion summarizing what is known about the growth pattern of fission yeast, including exponential, linear, and bilinear models proposed in previous papers, as well as the concept of NETO in the introduction part (see page 3). Moreover, we have emphasized that in practice, it is difficult to distinguish the exponential and bilinear growth patterns due to the stochasticity of growth dynamics and the relatively low temporal and spatial resolution of the data (see page 6).

4. *The author's state on page 10: "when N is very large ...". An illustration would be helpful, e.g. as part of Fig 3a,* $N = 600$.

Response: Fixed as suggested (see page 13, Figure 3(a), rightmost panel). Thank you.

5. *Why is the fission phase modelled as the end of the cell cycle, rather than the beginning? And why is this exponential? Atilgan ea (Curr Biol 2015) studied the new-end formation and the role of turgor pressure and might be relevant. If the size increase during fission is caused by a quick expansion of the new-end hemisphere, there is an upper bound on the added size during this phase, namely the size of a hemispherical end cap.*

Response: In the revised manuscript, we have clarified that (i) the rapid increase of cell length in the reshaping phase is the result of the rounding off of the new ends due to hydrostatic pressure, and (ii) the mean added size in this phase should roughly correspond to the size of the two hemispherical end caps. Moreover, we have also explained why we assume exponential growth with a higher growth rate in this phase (see pages 6-7). According to our data analysis, for all growth conditions, the added size and the duration in the reshaping phase are highly variable. For example, for the YE medium at 34°C, the mean and standard deviation of the added length in the reshaping phase are given by 1.590 ± 0.645 μm with a high coefficient of variation (CV) of 40.6%; the mean and standard deviation of the duration in the reshaping phase are given by 9.040 ± 3.274 minutes with a high CV of 36.2%. In our model, the generalized added size $\Delta_1 = V_d^\alpha - V_s^\alpha$ in the reshaping phase has an Erlang distribution with shape parameter N_1, whose CV is given by $1/\sqrt{N_1}$. When N_1 is small, the CV of the generalized added size is very large. This is consistent with experimental data.

6. *The authors state on page 17: "an interesting characteristic implied by the fission yeast data ...". This is unclear. More generally I find the distinction between model 1 and 2 unclear. Is there statistical support in the data for asymmetry? Or the data could purely be explained by stochastic partitioning.*

Response: In the revised manuscript, we have provided statistical support for asymmetry by carrying out the Z-test (see page 25). The deviation of p from 0.5 may result from the asymmetry in the position of the septum which is slightly nearer the new end. However, we cannot exclude the possibility that the

partitioning is actually symmetric and the deviation of p from 0.5 is an artifact due to the segmentation algorithm used in Reference [4], where the old-pole tips tend to be cut (Supplementary Fig. 3)

7. *The proportion of elongating cells seems a little lower than estimates of the proportion of cells in G2 phase (Mitchison and Nurse, J Cell Sci 1985; Carlson ea, J Cell Sci 1999).*

Response: In the revised manuscript, we have explained that the fraction of cell cycle in the elongation phase is not simply given by $r_0 = N_0/N$. This is because the transition rate between cell cycle stages is an increasing (power law) function of cell size, which means that earlier (later) stages have longer (shorter) durations. The real fraction w_0 of cell cycle in the elongation phase can be estimated approximately via Eq. (12). According to our estimation, the proportion of the elongation phase is about $72\% - 80\%$ for all growth conditions. This is consistent with the result in Reference [1] that the cell elongates during the first $\sim 75\%$ of the cell cycle.

8. *The table 2, only has N values but not N_0 and N_1, why is that? Could that be added for completeness.*

Response: Fixed as suggested (see page 19, Table 2). Thank you.
