# Supplementary material for: Characterizing non-exponential growth and bimodal cell size distributions in fission yeast: An analytical approach
Source: PLoS Comput Biol. 2022 Jan 18;18(1):e1009793. doi: 10.1371/journal.pcbi.1009793 (PMC8797179; doi:10.1371/journal.pcbi.1009793)
Supplement: Attachment — Submitted filename: Referee Response Letter.pdf [file pcbi.1009793.s007.pdf]

Response to Referee 1

Thank you for your valuable comments and suggestions which have helped us greatly in improving the quality of this paper. Below we provide a point-by-point response to each comment. All modifications made in the revised manuscript are shown in blue.

1. *Most of my comments have been adequately addressed. However I have one major issue that has come up in the process of revision that needs to be dealt with in some way. In my initial review I requested the authors plot the data they are using as "the relationship between birth size and* Δ*volume". This was because this is the classically defined relationship that describes size homeostasis in fission yeast. This has been reported many times and always shows a clear negative correlation with a slope of -0.7 to -1. Firstly the authors should also compute and report the slope data to allow comparison with prior publications. But more crucially it does appear from this new plot (Fig. 1d) that the data the authors are using shows a significantly different relationship to all other previously published results. This suggests there may be a critical problem with the authors choice of data and I would strongly encourage them to validate their model with another data set in which the anti-correlation between birth and growth volume is as expected.*

Response: In the revised manuscript, following your suggestions, we have illustrated the scatter plots of the birth length versus the added length for all the seven growth conditions and then computed the slopes of the regression lines (see Figure 5(e) on page 22). When computing the slope, we find that its value is significantly affected by outliers. For example, for YE at 34°C, removing a few outliers can change the slope remarkably from -0.35 to -0.55. To avoid the influence of outliers, we removed the outliers using the classical method in regression analysis and grouped the data into seven bins. Based on the lineage data, we find that the slope is typically -0.7 for EMM, which is consistent with the values reported in previous studies, while the slope is typically -0.5 for YE (see page 25). The reason why YE has a smaller slope is probably due to the fact that cells cultured in YE have faster growth rates, which give rise to weaker size control. Note that similar phenomenon has also been observed in *E. coli*, where an adder-like behavior was found in fast growth conditions, while a sizer-like behavior was found at low growth rates (see Reference [80]).

In the revised manuscript, we have not validated our model with another data set. This is because in order to fit our theory to experiments, we need high throughput time-course data of hundreds of cell lineages across many generations. We are sorry that we are not able to find other cell lineage data of fission yeast with so many data points.

Response to Referee 2

Thank you for your valuable comments and suggestions which have helped us greatly in improving the quality of this paper. Below we provide a point-by-point response to each comment. All modifications made in the revised manuscript are shown in blue.

1. *In Fig. 1. the cell length were better to be shown instead of area. Based on Table 2, the cell length must have been calculated correctly, however, the formula (area = length $\times$ width) given in the label to Fig. 1 is incorrect. The cell is a 3D cylinder (rather than a 2D rectangle).*

Response: In the revised manuscript, we have replaced cell area by cell length in Figure 1 (see page 4).

2. *The data given in Table 2 clearly indicate (corresponding to literature data) that bimodality is a result of that the "reshaping phase" strangely belongs here to the "old" cell cycle, meanwhile generally it is supposed to belong to the next one. The calculated septation size is about 13-14 micrometer, while the division size is about 15-16 micrometer. By contrast, what experimentalists in this field generally define as division size is only 13-14 micrometer. This fact should be explicitly given in the paper.*

Response: In the revised manuscript, we have further emphasized that the septation size defined in our paper is exactly the division size defined in previous papers since the reshaping phase is assumed to belong to the previous cell cycle (see page 24).

3. *In Table 2, the generation time (T) at 34 degrees C in EMM is given as 3.633 h. That seems to be too long for me, it should probably be 2.633 h or something like that.*

Response: We have double checked the data and found the mean doubling time in EMM at 34°C is indeed 3.633 h.

4. *I still cannot understand how the authors of this paper (and also of the Nakaoto-Wakamoto paper) could clearly define the timing of the end of the rounding off of the new cell poles (although they tried to do so in the revised version). No more want I to push the old hypothesis that the "reshaping phase" belongs to the new cell cycle, but I still have a feeling that the timing of the "onset" of this rounding off event is more clearly visible than that of its "finish". Please disprove my idea if possible; otherwise this problem is no more only theoretical, as it has experimental consequences as well.*

Response: In the revised manuscript, we have explained why the reshaping phase was assumed to belong to the previous cell cycle in the Nakaoto-Wakamoto paper (see the footnote on page 18). In fact, in the Nakaoto-Wakamoto paper, the authors converted fluorescence images into binary images, which were then used to identify cellular regions and track cell lineages. The cell division points were marked when the region of interest suddenly decreased more than 1.5-fold due to the clear separation of the two daughter cells. Since the two daughters did not form a clear boundary just after septation in binary images, the cell division point measured corresponds to the point at which the new cell end is formed and the two daughters clearly separate form each other.

5. *The above mentioned rounding off of the new cell poles is a consequence of the turgor pressure. Although sometimes it is called a hydrostatic pressure (even in textbooks, not only in this paper), I have a feeling that this is a bit incorrect.*

Response: In the revised manuscript, we have changed "hydrostatic pressure" to "turgor pressure" (see page 6).

6. *In the revised version, the authors correctly analysed the "activator accumulation" model, which may explain how size control mechanisms operate. However, there is another alternative hypothesis based in the "inhibitor dilution" model, which might also be correct, even in the case of the fission yeast cell cycle. I suggest that this should be mentioned in the paper.*

Response: In the revised manuscript, we have also mentioned the inhibitor dilution mechanism and the crucial role it plays in budding yeast (see pages 7-8).
